# Sustainable Shrimp Feeding: Digestibility of Defatted *Hermetia illucens* Meal by In Vitro and In Vivo Methods

**DOI:** 10.1155/anu/7323773

**Published:** 2025-11-23

**Authors:** Aurélien Dornic, Dominique Pham, Nelly Wabete, Nolwenn Callac, David Mazurais, Luca Donati, Marine Bézagu, José-Luis Zambonino-Infante

**Affiliations:** ^1^Innovafeed SAS, Nesle, France; ^2^LEMAR, University of Brest, Ifremer, CNRS, IRD, Plouzané, France; ^3^UMR 9220 ENTROPIE, Ifremer, IRD, Université de La Réunion, Université de la Nouvelle-Calédonie, Nouméa, New Caledonia

**Keywords:** black soldier fly, in vitro digestibility, in vivo digestibility, protein, shrimp

## Abstract

This study evaluated the digestibility of defatted black soldier fly larvae (BSFL, *Hermetia illucens*) meals as alternative protein sources to partially replace fish meal (FM) in blue shrimp (*Penaeus stylirostris*) diets. It also examined the relationship between in vivo and in vitro digestibility methods to support the development of a reliable in vitro approach. Two BSFL-based mixes were tested: one with a higher chitin content (H70), and the other with a lower chitin content and also presenting a more balanced essential amino acid profile (M70). Each replaced 60% of FM in experimental diets (H20 and M20, respectively) and were compared to a control diet containing no BSFL meal. Apparent digestibility coefficients (ADCs) were measured in vivo using chromium oxide (Cr_2_O_3_; 1%) as an inert marker. In vitro digestibility was assessed using the pH-stat method with shrimp hepatopancreas enzyme extracts. In vivo results showed that the M20 feed had significantly higher digestibility than the control (*p* <0.05), while no significant difference in protein ADC was observed among diets. In vitro results indicated that the H20 feed had lower digestibility than the control (*p* <0.05). The ranking of protein digestibility (Control ≥ M20 ≥ H20) was consistent between both methods. A satisfactory correlation was found between in vivo and in vitro protein digestibility (*R*^2^ = 0.691), which improved substantially after adjusting the in vitro assay temperature to match in vivo conditions (*R*^2^ = 0.864). These findings suggest that the pH-stat method is a promising tool for preliminary assessment of ingredient digestibility, more precisely protein digestibility, in shrimp diets. Moreover, industrial BSFL meal appears to be a viable protein source for replacing upto 60% of FM in shrimp feed formulations without compromising shrimp survival or digestibility.

## 1. Introduction

Over the past few decades, there has been a significant increase in the demand for seafood products. To address this growing demand, shrimp farming has expanded significantly worldwide, from 6.06 million tons (mT) in 2018 to 7.93 mT in 2022 [1]. The whiteleg shrimp, *Penaeus vannamei*, is the primary species produced, accounting for 6.8 mT in 2022 [1]. Other peneids, such as *Penaeus monodon* or *Penaeus stylirostris* are also raised in those areas. Compared to the whiteleg shrimp, these species are subject to less production because they are premium products and are more demanding in terms of protein requirements and water quality [2].

This expansion has intensified the need for aquaculture feeds. Historically, fish meal (FM) has been the main ingredient in aquaculture feeds due to its high protein and essential amino acid content [3]. However, the increased demand for FM and fish oil by industrial fisheries has put a strong pressure on ecosystems and led to the introduction of quota systems (particularly in South America). These led to a ceiling on the availability of FM, and therefore, the exploration of novel alternative protein sources, like plant-based proteins (wheat and soybean meal, wheat gluten, and corn meal). However, these protein sources often contain antinutritional factors, such as fiber or protease inhibitors [4], generally have less suited amino acid profile for carnivorous species and exhibit lower digestibility [4, 5]. Moreover, using terrestrial plant derivatives does not address environmental concerns, as it can exacerbate deforestation and eutrophication [6].

In this context, insect-based feed ingredients, in particular black soldier fly larvae (BSFL) meal, present a promising alternative. Indeed, insect rearing requires minimal space and water [7], has a small ecological footprint [8] and can convert various organic substrates into biomass. Insects' short life cycle and improved production techniques enable mass production on an industrial scale, addressing current environmental issues and making them an attractive, sustainable candidate for partially replacing FM in aquaculture feeds [9].

Insect meals offer high nutritional value, providing a rich source of proteins (50%–70%), lipids, vitamins, and minerals [10]. Black soldier fly exhibits an essential amino acid profile very similar to FM [11]. Studies evaluating BSFL meal in aquaculture have reported varying performances depending on the species, inclusion rate, and feed processing. While some studies found lower growth performance and digestibility with BSFL meal inclusion in turbot *Psetta maxima* [12], others reported satisfactory results in survival, growth performances and health parameters for various fish species, including trout [13, 14], salmon [15–17], catfish [18], or European seabass [19]. The meta-analysis of Hua [20] concluded that low or moderate dietary incorporation of insect meal has no impact on fish growth.

Concerning shrimp, studies have shown that insect meal can replace upto 20% of FM without any negative impact on growth performances in white leg shrimp *P. vannamei* [21–23] even upto 100% replacement of FM [24]. Insect meal incorporation can also improve growth, conversion index, immune status, and resistance to bacterial infection [25–27]. On the contrary, Li et al. [28] showed that the incorporation of two different insect meals (BSF meal and *Tenebrio molitor* meal) in the diet lead to a reduction in growth and digestibility.

The digestibility of alternative protein sources is mainly evaluated with an in vivo method. The method relies on the incorporation of an inert marker (chromium oxide [Cr_2_O_3_] or yttrium oxide) in the diets, animal feeding, feces collection for several weeks, and physico-chemical analysis. The in vivo method was proved to be suitable for aquaculture studies [29], but is costly, requires many animals, technical resources, space, and time. Therefore, it is not a suitable method for the screening and evaluation of multiple ingredients.

The development of a reliable and cost-effective in vitro method would increase the screening capacity of ingredients for the industry while reducing the need for in vivo testing, providing both economic benefits and improvements in animal welfare. However, to date, there is no reference method to evaluate the in vitro digestibility of aquafeed ingredients [30]. In shrimp, several studies [3, 31–35] are using the pH stat method to assess the protein digestibility. This method is faster, cheaper and allowed to screen more ingredients and the studies trend to show the feasibility of this method to evaluate the digestibility of ingredients and feeds in shrimp.

In this study, we evaluated the digestibility of different BSFL meal sources in blue shrimp (*P. stylirostris*) using both in vivo and in vitro methods. We assessed the relationship between both methods to determine if the pH stat method is suitable for shrimp studies.

## 2. Materials and Methods

### 2.1. Ethics Statement

Live blue shrimps *P. stylirostris* were used for the purpose of this study. For the in vivo digestibility study, the animals were reared under optimal conditions to maintain their welfare. For the hepatopancreas sampling (in vitro digestibility study), the animals were sacrificed and dissected after iced anesthesia to minimize their suffering.

### 2.2. Ingredients

Transformed insect protein from *Hermetia illucens* larvae, also called BSFL, was used for this study. BSFL meal was produced by Innovafeed SAS (Paris, France) from larvae reared on a Food Grade GMP + certificated substrate (composed mainly of starch industry byproducts). Mature live larvae (at their optimal growth and composition) were collected and sieved for the removal of excess substrate. They were then mixed with clean water, which allowed both the washing of remaining substrate and the slaughtering (water heating or blanching). Larvae were then drained, coarsely ground, and fat separation was achieved through dedicated equipment, which separated oil, water-soluble fraction (also called stick water [SW]), and solids based on density. The solids were then dried in a dryer. Sieving of this solid fraction through a 1.2 mm mesh led to the separation of two fractions of equivalent weight, respectively, designated as the “low-size fraction” (L) and “high-size fraction” (H). The water-soluble fraction (SW) in this work, was dried separately in a pilot scale atomization tower (Multi Stages Dryer Sicca Dania or equivalent equipment).

The digestibility of different fractions of these insect meals was tested with the in vitro method in order to determine the formulation of the experimental feeds for the in vivo digestibility trial. The proximal composition analysis of the standard shrimp feed ingredients were performed at the Laboratoire de Nouvelle-Calédonie (DAVAR), the nitrogen content was determined using the Kjeldahl technique and the results were multiplied by 5.6 to determine protein content. The proximal composition and the amino acids profile of the insect meals were analyzed by Eurofins laboratory (Laboratoire Nutrition Animal in Nantes). Their protein content was determined by sum of each amino acid content. Additionally, their chitin content was analyzed by Upscience laboratory (Saint-Nolff, France) by measuring glucosamine levels.

The different fractions of BSFL meal described above, alone or in combination, were tested in this work (Table 1).

### 2.3. In Vitro Digestibility

#### 2.3.1. Experimental Parameters

In vitro digestibility was measured by the pH-stat method with an automatic titrator (TritroLine 7000, SI Analytics). The experimental parameters were chosen to reflect the physiological shrimp digestive process and the rearing conditions as closely as possible. The pH was set to 8.0, the optimal pH of shrimp digestion and within the range of optimal values for several shrimp digestive enzymes [36–38], and the pH-stat method required alkaline conditions [30]. The temperature was set to 28°C, optimal for shrimp growth and representative of average farm temperature in New-Caledonia. The digestibilities of the experimental feeds were also evaluated at 22, 25 and 31°C to assess the impact of the temperature. The trial duration was set to 60 min, following previous studies using the pH-stat method [3, 35, 39]. It was determined that in vitro digestibility was repeatable for assays with a protein content ranging from 40 to 80 mg of protein [3, 34, 35].

#### 2.3.2. Enzyme Extract

Blue shrimps (208, mean weight = 23.04 ± 2.11 g) were harvested from rearing ponds at the Saint Vincent research station (Boulouparis, New-Caledonia). The animals were sacrificed and their hepatopancreas (the main digestive organ) were collected, pooled, and immediately frozen in pre-weighed dishes at −70°C. The tissues were grounded and homogenized in a 10 mM phosphate buffer, pH 7.4 (ratio 1:3 weight:volume, i.e., 1 g hepatopancreas to 3 mL buffer) with an ultra-Turrax, centrifuged twice (4°C, 10,000 rpm for 30 min) and the supernatant (containing soluble proteins including digestive enzymes) kept after removing the top lipid layer. The pH of the extract was adjusted to 8.0 with 1N NaOH, aliquoted by 2 mL and stored in the freezer at −20°C pending assays. The total soluble protein was determined by the Bradford method [40] with a BSA (bovine serum albumin) standard range. The activity of trypsin was measured according to the method of Holm et al. [41]. The specific activities of these enzymes were calculated according to the protein concentration of the extract.

#### 2.3.3. The pH-Stat Method

A 8 mL component sample (containing 40–80 mg protein; pH = 7.5) was added in the heated reaction chamber. When the target temperature was reached, a 45 min enzyme-free trial under continuous stirring was initiated. This step allowed for temperature stability verification and for the determination of the buffering capacity of the tested component by continuously adjusting the pH to 8.7 with 0.01N NaOH. Prior to the assay, the volume of enzyme extract was standardized following the methodology of Yasumaru and Lemos [42]. Preliminary trials were conducted using a standard protein substrate (80 mg casein) with varying volumes of the crude enzyme extract (50 to 1000 µL) to determine the minimum volume required for a robust and consistent hydrolytic response. This standardization resulted in an enzyme-to-substrate ratio of 6.1 mg of enzyme extract protein per 80 mg of protein substrate, which corresponded to a trypsin activity of 3.10 units for the same amount of protein substrate. Subsequently, this standardized volume (500 µL) was added to the reaction chamber. The titration was carried out during 60 min under permanent stirring, with the continuous adjustment of pH to 8.0 with 0.01N NaOH. The volume of NaOH added allowed us to evaluate the in vitro digestibility of the proteins by calculating the degree of hydrolysis (DH) as follows:

  $$\begin{array}{c} \text{DH }\left(\text{\% }\right)=\left(\left(V_{T}-V_{B}\right)\times N_{b}\times \frac{1}{\alpha }\times \frac{1}{M_{p}}\times \frac{1}{H_{\text{tot}}}\right)\times 100, \end{array}$$

where

*V*_T_ = volume of 0.01N NaOH added to maintain the pH at 8.0 in mL during the test;

*V*_B_ = volume of 0.01N NaOH added to maintain the pH 8.0 in mL during the buffering capacity test;

*N*_b_ = normality of the titrant (0.01);

*α* = dissociation coefficient of the α-amino groups at the temperature and pH studied (*α* = (10^pH − pK^)/(1 + 10^pH − pK^) [43]). At 28°C and pH = 8, *α* = 1.469;

*M*_p_ = mass of protein in the sample (g);

*H*_tot_ = total number of peptide bonds in the protein source (meq g protein^−1^) [44]. Calculate the content in mmol/g of each amino acid and sum up. If the amino acids profile is not available, the *H*_tot_ of the casein (8.22) can be used.

Studies showed that there is a strong correlation between the DH and the in vivo digestibility of the protein [3, 45], therefore, the DH can be used to estimate the digestibility of ingredients and feed in shrimps.

### 2.4. In Vivo Digestibility

#### 2.4.1. Experimental Diets

Different experimental diets were tested: one control feed with 32.75% FM (with no BSFL meal) and two experimental feeds with a 58% FM substitution (corresponding to 20% insect meal incorporation) by two BSFL defatted meals, either H70 or M70 (Table 2). The feeds were formulated to be isoproteic and isolipidic based on the proximate composition of the ingredients. 1% Cr_2_O_3_ was added as an inert marker to assess digestibility.

Ingredients were mixed with 30% distilled water to form a dough, then pelleted, dried for 20 h in a ventilated oven at 60°C and stored in a climate-controlled room.

#### 2.4.2. Feeding Trial

One hundred and sixty blue shrimps (average weight 21.95 ± 0.47 g) were transferred from the rearing pond to 10 (nine experimental tanks, one safety) 500 L circular resin-coated fiber tanks. Each one was supplied with sand-filtered seawater, air bubbler, and was equipped with a mesh (12 mm screen) to raise shrimps off the bottom, to avoid feces ingestion and to facilitate siphoning (cleaning and feces collection).

The day before the transfer, tanks were filled with a mixture of filtered seawater and fresh water to reach 26 PSU salinity, the iso-osmotic point in *P. stylirostris* [46] in order to minimize the handling stress in shrimp.

Fifteen shrimps were stocked on Day 0, and not fed, into each of the nine experimental tanks and 25 into the safety tank.

On Day 1, daily water renewal of 200% began and shrimps were fed on feeding trays with a commercial pellet (SICA, New Caledonia) twice daily (7:30 a.m. and 12:15 p.m.) for 2 h over a period of 5 days.

On Day 6, shrimps were fed with the control feed daily at a fixed feeding rate calculated as 4% of biomass. Feeding was divided into three rations: two in the morning which were left for 1 h, then removed and one in the afternoon was distributed as a single ration and left for 1 h and 30 min. Shrimps were fed in this way throughout the experiment. During this 5-days period, 2 siphoning operations were carried out daily after each feeding to remove food scraps and waste (exuviae, wastes, and feces). This practice also helped animals become accustomed to the operations in the tanks. During both acclimation periods, any dead animals in the experimental tanks were replaced with shrimps from the safety tank.

On Day 11, the feeding trial was started. Animals were fed ad libitum with the experimental diets (in triplicate, randomly assigned) using feeding trays as described above for a period of 9 weeks. Throughout this period, the tanks were cleaned after each feeding (leftovers, exuviae, and feces removed). At least 1 h after cleaning (time for animals to defecate), the feces were collected by siphoning through a 500 µm mesh, rinsed with distilled water, and stored in a jar in the freezer at −20°C. Feces collected from each tank were pooled in the same jar throughout the experiment.

Temperature and salinity were measured twice daily (7:30 a.m. and 3:00 p.m.) using a conductivity meter (WTW modèle COND3320), and oxygen concentration was measured weekly using an oximeter (Oxyguard Handy Polaris).

#### 2.4.3. Feeds and Feces Analysis

The chromium and nitrogen content of the feeds and the feces were analyzed by the LAMA of Noumea (Laboratoire des Moyens Analytiques, UAR IMAGO, certified ISO 9001). Nitrogen content was determined using the Kjeldahl technique and the results were multiplied by 5.6 [47] to evaluate the protein content of ingredients and feeds. The chromium content was analyzed using alkaline fusion with ICP-OES analysis.

Dry matter was determined by weighing samples before and after oven drying for 24 h at 105°C. Mineral matter concentrations were measured after 5 h in a muffle oven at 550°C for both feeds and feces.

Total lipid content was determined using the Folch et al. [48] micro-method, based on cold centrifugal extraction with a mixture of apolar (dichloromethane) and polar (methanol) solvents in a 2:1 ratio.

#### 2.4.4. Coefficients of Digestibility

The results of the diets and the feces compositions were used to calculate the apparent digestibility coefficients (ADCs) of the experimental feeds (ADC_D_) and the protein (ADC_P_). These coefficients are calculated using the formulas provided by Wilson and Poe [49] and Guillaume et al. [50]:

  $$\begin{array}{c} {\text{ADC}}_{D}\;\left(\%\right)=100-100\times \left(\frac{{\text{Cr}}_{D}}{{\text{Cr}}_{F}}\right), \end{array}$$

  $$\begin{array}{c} {\text{ADC}}_{P}=100-100\times \left(\frac{{\text{Cr}}_{D}}{{\text{Cr}}_{F}}\times \frac{P_{F}}{P_{D}}\right), \end{array}$$

where Cr_D_ = the chromium content in the diet; Cr_F_ = the chromium content in the feces; *P*_D_ = the protein content in the diet; *P*_F_ = the protein content in the feces.

### 2.5. Statistical Analysis

Data were analyzed with R-studio software (RStudio 2023.06.1 and R4.2.2) with tidyverse, ggpubr, rstatix, outliers, mgcv, car, dplyr, and tibble packages [51–58]. Data were analyzed using ANOVA or Kruskal–Wallis tests (depending on the distribution of the data) and post hoc mean rank tests (Tukey for ANOVA and Dunn for Kruskal–Wallis; *p*-value <0.05).

## 3. Results

### 3.1. Characterization of Enzyme Extract

The protein concentration of the enzyme extract was measured at 12.21 ± 0.10 mg/mL and exhibited a trypsin specific activity of 0.508 ± 0.028 U/mg of protein.

### 3.2. In Vitro Digestibility of the Meals

Different insect meals, mixes, and a FM were tested with the pH-stat method to evaluate their digestibility (Table 3). The temperatures of the tests were not different (mean temperature = 28.09 ± 0.08°C) and there was no significant difference between the protein quantity of the tests except between the SW and the L meal (Dunn test, *p*-value = 0.005), however, it remains within the range of 40–80 mg of protein. The DH of the L and H meals were lower from that of the SW (ANOVA, *p* ~ 0.001 and ~ 0.001, respectively).

The chosen BSFL meals were compared to the FM. There were significant differences between the in vitro digestibility of the FM compared to H70 and M70 (ANOVA; Table 3).

### 3.3. In Vitro Digestibility of Experimental Feeds

There was no difference either in the temperature or the protein quantity of the tests (mean temperature = 28.08 ± 0.04°C). The digestibility of the H20 feed was lower than the control (*p*-value = 0.034; Table 4)

**Figure 1:**
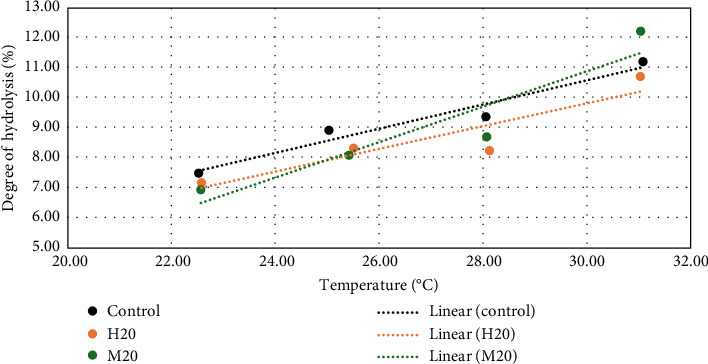
Evolution of the in vitro digestibility of the three experimental feeds by varying the temperature (at 22.5, 25.0, 28.0, and 31.0°C). The equation of the linear regression is *y* = 0.004*x* – 0.015 with a *R*^2^ = 0.95 for the control diet; *y* = 0.0038*x* – 0.0155 with a *R*^2^ = 0.84 for the H20 diet and *y* = 0.0059*x* – 0.068 with a *R*^2^ = 0.88 for the M20 diet.

The DH of the experimental feeds at various temperatures was tested to evaluate the linear relationship between the in vitro protein digestibility and the temperature (Figure 1). All the *R*^2^ for this relationship were greater than 0.83.

### 3.4. Feed Compositions

The analysis of the experimental feeds showed that there was no difference in protein concentration (average protein concentration = 36.29% ± 1.24%, Table 5), nor in the lipid and chromium contents of the treatments. It should be noted that there is no difference in composition between the control group and M20. The analysis also showed that the dry matter content of the H20 feed was slightly higher than the control feed. The mineral matter content of the H20 feed was slightly lower than the control feed.

### 3.5. Feeding Trial

The tank parameters (temperature, oxygen, and salinity) were very stable throughout the experiment (mean temperature = 29.38 ± 0.03°C, mean salinity = 36.98 ± 0.01 PSU, and mean oxygen concentration = 5.92 ± 0.01 mg/mL).

There was no difference of the survival rate between the treatments (mean survival rate = 80.00% ± 11.55%).

The digestibility of the individual ingredients was not calculated because we chose to focus on the whole feed and protein digestibility to compare the in vivo and in vitro methods.

The ADC_D_ of M20 feed was higher than the control feed (ANOVA, *p* = 0.003). There was no difference in the ADC of the protein (ADC_P_) among the treatments. The digestibility results are shown in Table 6.

### 3.6. Comparison Between the In Vitro and the In Vivo Methods

The DH of the protein was compared to the ADC of the protein (ADC_P_). The ranking of the digestibility of the protein was conserved between the methods: Control ≥ M20 ≥ H20.

**Figure 2:**
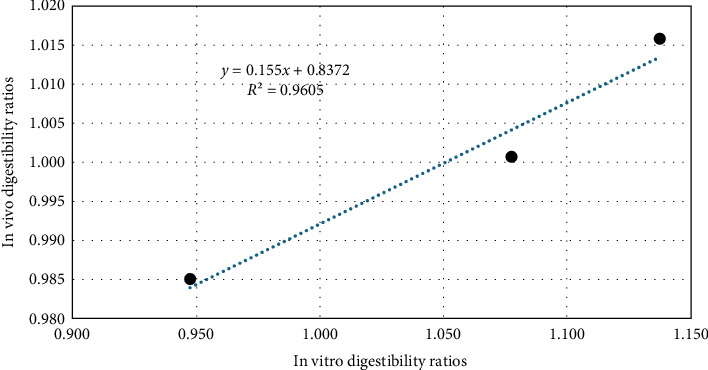
Relationship between the calculated ratios of in vivo and in vitro digestibility methods.

The ratios between the treatments for both methods were calculated and compared (Table 7 and Figure 2). A linear relationship with a *R*^2^ = 0.960 was obtained between the in vitro and the in vivo methods.

**Figure 3:**
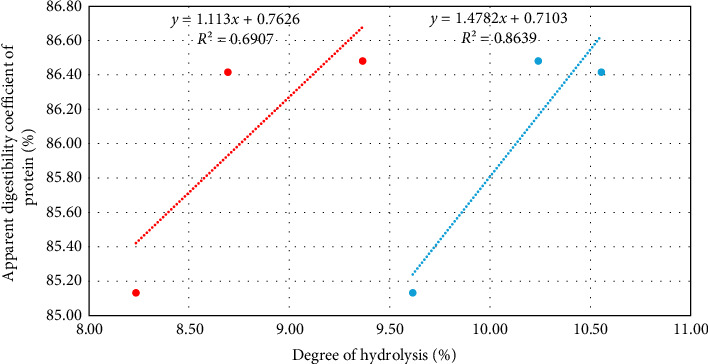
Relationships between the in vitro and in vivo digestibility results. In red the linear relationship using in vitro results at 28.08 ± 0.04°C; in blue the linear relationship using the in vitro results adjusted to 29.38 ± 0.03°C.

The relationship between the results obtained was also evaluated (Figure 3). A linear relationship with a *R*^2^ = 0.691 was obtained on the raw values.

The in vivo and in vitro trials were conducted at 29.38 ± 0.03°C and 28.08 ± 0.04°C, respectively. By correcting the in vitro digestibility values to the temperature of the in vivo digestibility with the linear relationships established (Figure 1), we obtained a corrected linear relationship with a *R*^2^ of 0.864 between the estimated DG and the ADC

## 4. Discussion

The purpose of this study was to evaluate the potential use of the BSFL meal as a partial replacement of FM in the nutrition of farmed shrimps. However, given that the quality of BSFL meals products is still not standardized, unlike the FM (which has standard quality grades), their nutritional values could vary from one source to another, as well in different fractions of the same product. Digestibility is a key parameter in shrimp feed formulation. As it is ethically and economically complicated to test all the products by an in vivo approach, it appeared necessary to develop a rapid and reliable in vitro digestibility technique for screening the products, and to confirm the results of only the best ingredients using in vivo digestibility method.

BSFL meal is a promising protein source with an essential amino acid profile similar to FM, though it may be limiting in lysine and methionine for shrimp [59]. Furthermore, it contains chitin, a component known to enhance immune response [60] and essential for shrimp growth via molting [61–63].

Research on the effects of BSFL meals on several biological parameters in shrimps has intensified over the past few years, yielding diverse results depending on the meal composition, inclusion rate, and feed composition.

Chen et al. [23] showed that FM can be replaced by BSF meal up to 10%, improving survival in challenge tests in *P. vannamei* without impacting growth performances. They used a nondefatted meal (with 35.17% protein and 32.6% lipid), which may have limited its incorporation into the diet. Cummins et al. [22], who used a defatted BSF meal containing 52.03% protein and 15.10% lipid, found that FM can be replaced by upto 25% without affecting growth performances. Similarly, Richardson et al. [64] reported improved growth performances in *P. vannamei* by replacing 30% of FM with defatted BSFL meal (~60% protein and 7% lipid).

Li et al. [28] showed that incorporating 30% BSF meal into the diet resulted in reduced growth performances and digestibility of the dry matter and protein (ADC_P_ of 91.67% ± 0.31% and 72.41% ± 0.47% obtained for the control diet and the BSFL meal diet, respectively). Conversely, Shin et al. [27] found that, at the same inclusion rate, BSF meal had no impact on diet and protein digestibility (ADC_P_ of 89.2% ± 1.62% and 85.1% ± 5.58% obtained for the control diel and the BSFL meal diet, respectively), and significantly improved growth and health performance. This discrepancy could be attributed to the composition of the BSF meal used in each study. Li et al. [28] employed a nondefatted BSF meal containing 32.6% lipids, whereas Shin et al. [27] used a defatted BSF meal with 17.4% lipids. Consequently, the final diet lipid content varied between 14.1% and 11.0%, respectively. Previous studies [65, 66] recommend a daily lipid intake for *P. vannamei* between 6% and 12%. Exceeding these levels may lead to lipid accumulation, potentially limiting energy metabolism and negatively affecting survival, growth performances and digestibility. Concerning the effect of lipids on the DH measurement, it should be noted that presence of lipids could potentially negatively interfere with protein hydrolysis by creating a physical barrier, depending on the quantity and the size of the droplets [67, 68]. Also, the pH-stat method can be adapted to measure lipid hydrolysis (under different conditions than protein hydrolysis) and lipids may impact the reaction by consuming alkali if lipase is present in the enzyme extract, thus, overestimating the results. That is why the lipid content of the matrix needs to be controlled to minimize its impact, even more in our case using enzyme extract.

These studies reveal that depending on the matrix used, the effects of insect meal on shrimp can be variable, even contradictory. Therefore, it seems quite relevant to have a reliable and easy-to-implement in vitro approach that can inform the choice of formulations to be tested in vivo.

In vitro approaches have traditionally relied on enzymatic reactions to evaluate the digestibility. Early studies assessed digestibility through enzymatic hydrolysis of feeds [36, 69]. Alternatively, methods like pH-stat and pH-drop measure pH changes in the solution. Ezquerra et al. [31] found that both methods are suitable for assessing shrimp in vitro digestibility, with the pH-stat method yielding better results due to a strong correlation between the DH and ADC of protein (ADC_P_) in *P. vannamei*. Subsequent studies have confirmed the feasibility of this approach, showing a strong correlation between the DH and the ADC_P_ [3, 44] or between the DH and the growth performance [34] in whiteleg shrimp. Given the simpler digestive system of crustaceans (one phase of digestion), this method is particularly suitable for shrimps compared to fish species with functional stomachs [30]. The pH-stat method has been widely used in various studies to assess the digestibility of ingredients [35, 70]. The approach can be set up either using commercial enzyme mix or with enzyme extract from living animals. Commercial enzyme mixes are often derived from mammalian sources (porcine and bovine). Lazo et al. [71] concluded that this commercial mix and porcine trypsin are suitable for evaluating digestibility in *P. vannamei* using the pH-drop method. However, those mixes may have different optimal pH and temperature conditions. Lemos et al. [33] found differences when comparing the pH-stat method using enzyme extracts from *Farfantepenaeus paulensis* to a commercial mix (from Satterlee et al., [72]). Most assays on crustaceans use enzyme extracts from the digestive gland (hepatopancreas) because they more accurately represent the enzymatic composition of the animals. Species-specific extracts should be prepared taking into account variability in weight, life stage, and health status. These extracts contain co-factors, and all the enzymes involved in the digestive hydrolysis [33, 73, 74], which are not present in commercial enzyme mixes.

We chose to use enzyme extracts from blue shrimp from our research facility to ensure greater representativeness since no study exists on in vitro digestibility in *P. stylirostris*.

Using the pH-stat method, we evaluate the digestibility of various BSFL meal products compared to a control feed. The in vitro digestibility of the M70 mix, which has a more balanced essential amino acids profile, is higher than that of the H70 mix, which contains more chitin. A similar, though nonsignificant, pattern is observed between the M70-based feed and the H70-based feed. The latter has a slightly lower digestibility than the control feed. While peneids can digest chitin [75], the efficiency may vary by species. For instance, *P. vannamei* can digest chitin as long as dietary content does not exceed 2% [75]. Shin et al. [27] also noted that *P. vannamei* can tolerate dietary chitin from BSF meal if levels are not excessive (0.85% chitin in the BSFL meal feed in the study). Additionally, *P. monodon* shrimp fed a 5% chitin feed exhibited higher weight gain than those on a control diet [76]. Based on the analyses of the meals provided by Innovafeed, we estimated that the chitin levels in our feeds were moderate, with H20 (1.78%) containing 40% more chitin than M20 (1.26%). While these levels should be suitable for shrimp nutrition, further confirmation is needed specifically for *P. stylirostris*.

We wanted to test different mixes, both with similar digestibility to a control feed, with varying compositions. The formulated feeds contain different amounts of chitin, since this compound, in addition to its nutritional value for shrimp, could also have functional properties, such as immunostimulation [59]. However, it could also act as an antinutritional factor if its level is excessive. The feed composed with the M70 mix looks promising with the in vitro approach.

The method used to assess digestibility in shrimp with Cr_2_O_3_ as an inert marker is a standard, commonly used, and well referenced approach, provided the marker's inclusion remains low [29, 77, 78].

In our study, both BSFL meals were incorporated at a 20% rate into the diets, without any adverse effects on shrimp survival. The digestibility of the feeds (above 70%) and their protein digestibility (>85% for all) were satisfactory. The ranking of the digestibility of the experimental feeds was: M20 ≥ H20 ≥ Control. Our results can be compared to those obtained in P. vannamei by Shin et al. [27], where the ranking of ADC_D_ is BSFL diet ≥ Control. The authors obtained ADC_P_ results similar to our study (89.2% ± 1.62% and 85.1% ± 5.58% for the control diel and the BSFL meal diet, respectively, while we found 86.48% ± 0.87% for the control diet and 85.13% ± 1.81% and 86.41% ± 1.50% for both BSFL meal diets). However, our results showed lower digestibility of the control diet compared to Shin and Lee study (70.31% ± 2.53% and 78.1% ± 4.20%, respectively). Since the proximate composition of the control diets are quite similar and the other results (protein digestibilities and BSFL meal diets digestibilities) are comparable, it appears unlikely that this difference comes from the use of different species (*P. vannamei* and *P. stylirostris*) or the slight variation in the composition of the feeds. Thus, it might be explained by either the differences in the formulation of the control diets or the size of the studied animals.

Our findings indicate that the in vivo digestibility of the whole feed ranks as follows: M20 ≥ H20 ≥ Control. However, when focusing on protein digestibility, assessed via ADC_P_ (in vivo) and the DH (in vitro), who both evaluate the protein digestibility, the ranking shifts to: Control ≥ M20 ≥ H20.

The differing digestibility rankings highlight the complementary nature of each method's focus. The ADC_D_, a global in vivo measure of feed assimilation, indicates that the experimental diets, containing BSFL meals (M20 and H20), were slightly better utilized overall than the control, likely due to additional digestible components in the BSFL meal enhancing total nutrient absorption.

In contrast, ADC_P_ and DH, which specifically assess protein digestibility, reveal a ranking inversely correlated with dietary chitin content (Control ≥ M20 ≥ H20). This suggests that chitin may act as a physical barrier, impairing protein digestion, and explain why the control diet exhibited the highest protein digestibility.

Far from contradictory, these results offer a comprehensive physiological picture: the M20 feed (and M70 ingredient), demonstrates a balanced nutritional profile for *P. stylirostris*, with consistent protein digestibility ranking across in vivo and in vitro methods.

Additionally, the ratios between feeds are maintained across both methods supporting the reliability of our in vitro method. The in vitro data showed a difference between the control feed and the H20 feed that was not observed in the in vivo approach, likely due to environmental and physiological conditions affecting digestibility [76]. The linear relationship between the methods is satisfactory (*R*^2^ = 0.691). A stronger correlation was observed after adjusting in vitro values to the temperature of the in vivo digestibility (*R*^2^ = 0.864). This result highlights the importance of temperature in ensuring greater representativeness, without minimizing the potential role of other abiotic parameters, such as pH, ionic strength, and salinity, which were not tested in this study. It also suggests that the pH-stat method is suitable for assessing digestibility as long as the tested conditions, particularly the temperature, closely reflect those of the in vivo method.

## 5. Conclusion

Our study demonstrates that the pH-stat method is a promising approach for initially assessing the digestibility of ingredients and feeds in shrimps, provided that the experimental parameters are well established to ensure greater representativeness. However, additional data from both methods, on feeds with varying composition, needs to be acquired in order to develop a reliable equation between the in vitro and in vivo methods. Also, characterizing and standardizing the enzyme extract from blue shrimp hepatopancreas could be a relevant step toward formulating a reliable commercial enzyme mix, thereby enhancing the reproducibility of the in vitro method. Additionally, we show that industrial BSFL meal can partially replace FM in the diet of *Penaeus stylirostris* without negatively impacting survival or digestibility. What now remains to be examined is the functional potential of these BSFL meals on the physiology and robustness of shrimp, notably its immunomodulatory capabilities.

## Figures and Tables

**Table 1:** Description of the insect meals and mixes.

Abbreviation	Relative composition of the mix	Protein content (%)	Description
SW	—	34.94	Water-soluble fraction (stick water) dried by atomization
L	—	61.15	Low-size fraction obtained through sieving of the BSFL solid fraction. This low-size fraction is mainly characterized by a lower chitin content than the unsieved fraction
H	—	53.25	High-size fraction obtained through sieving of the BSFL solid fraction. This high-size fraction is mainly characterized by a higher chitin content than the unsieved fraction
H70	H + SW (70:30)	47.76	Mix of the high-size fraction with SW at a mass ratio (on dry matter) of 70:30
M	L + H (50:50)	57.20	Mix of the high-size fraction with the low-size fraction at a mass ratio (on dry matter) of 50:50 to reconstitute the BSFL initial solid fraction
M70	M + SW (70:30)	50.52	Mix of the M fraction with SW at a mass ratio (on dry matter) of 70:30 This product has better essential amino acids profile and is representative of the industrial product solid to water soluble fraction ratio

*Note:* All the fractions are produced and provided by Innovafeed SAS, Nesle, France.

**Table 2:** Formulation of the experimental feeds.

Ingredients (%)	Control	H20	M20
Fish meal^a^	32.75	13.75	13.75
H70	0	20	0
M70	0	0	20
Soybean meal^b^	16.25	16.25	16.25
Wheat meal^c^	33.75	31.25	31.25
Wheat gluten^d^	8.25	8.25	8.25
Fish oil	4.4	5.9	5.9
Dicalcium phosphate	2	2	2
Vitamin premix^e^	0.5	0.5	0.5
Mineral premix^f^	0.8	0.8	0.8
Vitamin C	0.3	0.3	0.3
Chromium oxide^g^	1	1	1

^a^Peruvian fish meal contains 61.9% crude protein.

^b^Soybean meal (GMO free) contains 52.5% crude protein.

^c^Wheat meal contains 13.3% crude protein.

^d^Wheat gluten contains 76% crude protein.

^e^Vitamin premix, from SICA, New Caledonia (per kg of diet): vitamin A, 800 IU; vitamin D, 400 IU; vitamin E, 40 mg; menadione (vitamin K), 8 mg; thiamin (vitamin B1), 8 mg; riboflavin (vitamin B2), 8 mg; niacin (vitamin B3), 40 mg; calcium pantothenate (vitamin B5), 22 mg; pyridoxine (vitamin B6), 10 mg; folate (vitamin B9), 2 mg.

^f^Mineal premix, from SICA, New Caledonia (per kg of diet): copper, 16 mg; zinc, 41 mg; manganese, 12 mg; magnesium, 56 mg; cobalt, 0.02 mg; iodine, 2 mg; selenium, 0.08 mg.

^g^Chromium(III) oxide, powder, ≥98%, Sigma-Aldrich, USA.

**Table 3:** Parameters and results of the in vitro digestibility tests.

Meals	Protein quantity (mg)	Temperature of the test (°C)	Degree of hydrolysis (%)
FM	73.28 ± 1.35^ab^	28.08 ± 0.12^a^	8.32 ± 0.49^a^
SW	56.93 ± 1.29^b^	28.01 ± 0.08^a^	6.67 ± 0.26^b^
L	80.81 ± 0.29^a^	28.00 ± 0.35^a^	3.59 ± 0.11^d^
H	75.25 ± 0.62^ab^	28.16 ± 0.11^a^	5.03 ± 0.23^c^
H70	68.39 ± 1.33^ab^	28.22 ± 0.10^a^	6.04 ± 0.15^b^
M70	67.91 ± 3.63^ab^	28.08 ± 0.04^a^	6.13 ± 0.22^b^

*Note:* Values are presented as mean ± standard deviation (*n* = 3 for each meal). Significant differences (ANOVA or Kruskal–Wallis and associated post hoc mean rank tests, *p*-value < 0.05) are annotated with superscript lowercase letters. H, high-size fraction of BSFL meal; H70, mix of the high-size fraction with SW at a mass ratio of 70:30; L, low-size fraction of BSFL meal; M70, mix of the high and low size fraction (50:50) with SW at a mass ratio of 70:30.

Abbreviations: BSFL, black soldier fly larvae; FM, fish meal; SW, stick water.

**Table 4:** In vitro digestibility of the experimental feeds (degree of hydrolysis in %).

Diet	Degree of hydrolysis (%)
Control	9.36 ± 0.53^a^
H20	8.23 ± 2.53^b^
M20	8.69 ± 2.53^ab^

*Note:* Values are presented as mean ± standard deviation (*n* = 3 for each meal). Significant differences (ANOVA or Kruskal–Wallis and associated post hoc mean rank tests, *p*-value < 0.05) are annotated with superscript lowercase letters. H20, feed formulated with the mix of stick water with the high-size fraction of black soldier fly larvae (BSFL) meal (mass ratio of 30:70) incorporated at a 20% rate; M20, feed formulated with the mix of stick water with the low and high-size fractions of BSFL meal (mass ratio of 30:35:35) incorporated at a 20% rate.

**Table 5:** Proximate composition of experimental feeds.

Composition (%)	Control	H20	M20
Protein content	37.68 ± 0.71^a^	35.91 ± 1.37^a^	35.28 ± 1.69^a^
Lipid content	8.82 ± 0.24^a^	9.46 ± 0.82^a^	9.39 ± 0.49^a^
Chromium content	0.65 ± 0.02^a^	0.60 ± 0.05^a^	0.58 ± 0.08^a^
Dry matter	94.51 ± 0.02^b^	95.72 ± 0.06^a^	95.47 ± 0.01^ab^
Mineral matter	12.95 ± 0.05^a^	9.95 ± 0.04^b^	10.27 ± 0.07^ab^

*Note:* Values are presented as mean ± standard deviation. Significant differences (*p*-value < 0.05) are annotated with superscript lowercase letters. H20, feed formulated with the mix of stick water with the high-size fraction of black soldier fly larvae (BSFL) meal (mass ratio of 30:70) incorporated at a 20% rate; M20, feed formulated with the mix of stick water with the low and high-size fractions of BSFL meal (mass ratio of 30:35:35) incorporated at a 20% rate.

**Table 6:** In vivo digestibility results.

Digestibility (%)	Control	H20	M20
ADC_D_	70.31 ± 2.53^b^	73.73 ± 3.00^ab^	75.89 ± 1.80^a^
ADC_P_	86.48 ± 0.87^a^	85.13 ± 1.81^a^	86.41 ± 1.50^a^

*Note:* The apparent digestibility coefficients of the feeds (ADC_D_) and the protein (ADC_P_) of the experimental diets. Significant differences (ANOVA and post hoc mean rank tests, *p*-value <0.05) are annotated with superscript lowercase letters. For each treatment *n* = 6.

**Table 7:** Calculated ratios of the digestibility results for both in vivo and in vitro methods.

Ratio	In vitro	In vivo
Control/H20	1.137	1.016
Control/M20	1.077	1.001
H20/M20	0.947	0.985

*Note:* The ratios were calculated using the average digestibility of each treatment for both methods.

## Data Availability

The raw data that support the findings of this study are available on request from the corresponding author. The data are not publicly available due to industrial confidentiality.

## References

[1] FAO (2024). The State of World Fisheries and Aquaculture 2024.

[2] (2004). *Introductions and Movement of Penaeus vannamei and Penaeus stylirostris in Asia and the Pacific*.

[3] Lemos D., Lawrence A. L., Siccardi A. J. (2009). Prediction of Apparent Protein Digestibility of Ingredients and Diets by In Vitro pH-Stat Degree of Protein Hydrolysis With Species-Specific Enzymes for Juvenile Pacific White Shrimp *Litopenaeus Vannamei*. *Aquaculture*.

[4] Krogdahl Å., Penn M., Thorsen J., Refstie S., Bakke A. M. (2010). Important Antinutrients in Plant Feedstuffs for Aquaculture: An Update on Recent Findings Regarding Responses in Salmonids. *Aquaculture Research*.

[5] Glencross B. D., Booth M., Allan G. L. (2007). A Feed is Only as Good as its Ingredients? A Review of Ingredient Evaluation Strategies for Aquaculture Feeds. *Aquaculture Nutrition*.

[6] Mastoraki M., Mollá Ferrándiz P., Vardali S. C. (2020). A Comparative Study on the Effect of Fish Meal Substitution With Three Different Insect Meals on Growth, Body Composition and Metabolism of European Sea Bass (*Dicentrarchus Labrax* L.). *Aquaculture*.

[7] van Huis A. (2013). Potential of Insects as Food and Feed in Assuring Food Security. *Annual Review of Entomology*.

[8] Oonincx D. G. A. B., de Boer I. J. M., Sword G. A. (2012). Environmental Impact of the Production of Mealworms as a Protein Source for Humans—A Life Cycle Assessment. *PLoS ONE*.

[9] PhI Cé P. V., Walraven M., Bézagu M., Lefranc M., Ray C. (2020). Industrial Symbiosis in Insect Production—A Sustainable Eco-Efficient and Circular Business Model. *Sustainability*.

[10] Barroso F. G., de Haro C., Sánchez-Muros M. J., Venegas E., Martínez-Sánchez A., Pérez-Bañón C. (2014). The Potential of Various Insect Species for Use as Food for Fish. *Aquaculture*.

[11] Bruni L., Pastorelli R., Viti C., Gasco L., Parisi G. (2018). Characterisation of the Intestinal Microbial Communities of Rainbow Trout (*Oncorhynchus Mykiss*) Fed With *Hermetia Illucens* (Black Soldier Fly) Partially Defatted Larva Meal as Partial Dietary Protein Source. *Aquaculture*.

[12] Kroeckel S., Harjes A.-G. E., Roth I. (2012). When a Turbot Catches a Fly: Evaluation of a Pre-Pupae Meal of the Black Soldier Fly (*Hermetia Illucens*) as Fish Meal Substitute—Growth Performance and Chitin Degradation in Juvenile Turbot (Psetta Maxima). *Aquaculture*.

[13] Renna M., Schiavone A., Gai F. (2017). Evaluation of the Suitability of a Partially Defatted Black Soldier Fly (*Hermetia Illucens* L.) Larvae Meal as Ingredient for Rainbow Trout (*Oncorhynchus Mykiss* Walbaum) Diets. *Journal of Animal Science and Biotechnology*.

[14] Sealey W. M., Gaylord T. G., Barrows F. T. (2011). Sensory Analysis of Rainbow Trout, *Oncorhynchus Mykiss*, Fed Enriched Black Soldier Fly Prepupae, *Hermetia Illucens*. *Journal of the World Aquaculture Society*.

[15] Lock E. R., Arsiwalla T., Waagbø R. (2016). Insect Larvae Meal as an Alternative Source of Nutrients in the Diet of Atlantic Salmon (*Salmo Salar*) Postsmolt. *Aquaculture Nutrition*.

[16] Belghit I., Liland N. S., Gjesdal P. (2019). Black Soldier Fly Larvae Meal Can Replace Fish Meal in Diets of Sea-Water Phase Atlantic Salmon (*Salmo Salar*). *Aquaculture*.

[17] Weththasinghe P., Hansen J., Nøkland D., Lagos L., Rawski M., Øverland M. (2021). Full-Fat Black Soldier Fly Larvae (*Hermetia Illucens*) Meal and Paste in Extruded Diets for Atlantic Salmon (*Salmo Salar*): Effect on Physical Pellet Quality, Nutrient Digestibility, Nutrient Utilization and Growth Performances. *Aquaculture*.

[18] Fawole F. J., Adeoye A. A., Tiamiyu L. O., Ajala K. I., Obadara S. O., Ganiyu I. O. (2020). Substituting Fishmeal With *Hermetia Illucens* in the Diets of African Catfish (*Clarias Gariepinus*): Effects on Growth, Nutrient Utilization, Haemato-Physiological Response, and Oxidative Stress Biomarker. *Aquaculture*.

[19] Magalhães R., Sánchez-López A., Leal R. S., Martínez-Llorens S., Oliva-Teles A., Peres H. (2017). Black Soldier Fly (*Hermetia Illucens*) Pre-Pupae Meal as a Fish Meal Replacement in Diets for European Seabass (*Dicentrarchus Labrax*). *Aquaculture*.

[20] Hua K. (2021). A Meta-Analysis of the Effects of Replacing Fish Meals With Insect Meals on Growth Performance of Fish. *Aquaculture*.

[21] Panini R. L., Freitas L. E. L., Guimarães A. M. (2017). Potential Use of Mealworms as an Alternative Protein Source for Pacific White Shrimp: Digestibility and Performance. *Aquaculture*.

[22] Cummins V. C., Rawles S. D., Thompson K. R. (2017). Evaluation of Black Soldier Fly (*Hermetia Illucens*) Larvae Meal as Partial or Total Replacement of Marine Fish Meal in Practical Diets for Pacific White Shrimp (*Litopenaeus Vannamei*). *Aquaculture*.

[23] Chen Y., Chi S., Zhang S. (2021). Evaluation of the Dietary Black Soldier Fly Larvae Meal (*Hermetia illucens*) on Growth Performance, Intestinal Health, and Disease Resistance to *Vibrio parahaemolyticus* of the Pacific White Shrimp (*Litopenaeus vannamei*). *Frontiers in Marine Science*.

[24] Rahimnejad S., Hu S., Song K. (2019). Replacement of Fish Meal With Defatted Silkworm (*Bombyx Mori* L.) Pupae Meal in Diets for Pacific White Shrimp (*Litopenaeus Vannamei*). *Aquaculture*.

[25] Choi I. H., Kim J. M., Kim N. J. (2017). Replacing Fish Meal by Mealworm (*Tenebrio molitor*) on the Growth Performance and Immunologic Responses of White Shrimp (*Litopenaeus vannamei*). *Acta Scientiarum. Animal Sciences*.

[26] Motte C., Rios A., Lefebvre T., Do H., Henry M., Jintasataporn O. (2019). Replacing Fish Meal With Defatted Insect Meal (Yellow Mealworm *Tenebrio Molitor*) Improves the Growth and Immunity of Pacific White Shrimp (*Litopenaeus Vannamei*). *Animals*.

[27] Shin J., Lee K.-J., Dawood M. A. O. (2021). Digestibility of Insect Meals for Pacific White Shrimp (*Litopenaeus Vannamei*) and Their Performance for Growth, Feed Utilization and Immune Responses. *PLoS ONE*.

[28] Li X., Chen Y., Zheng C. (2022). Evaluation of Six Novel Protein Sources on Apparent Digestibility in Pacific White Shrimp, *Litopenaeus Vannamei*. *Aquaculture Nutrition*.

[29] Smith D. M., Tabrett S. J. (2004). Accurate Measurement of In Vivo Digestibility of Shrimp Feeds. *Aquaculture*.

[30] Moyano F. J., Saénz de Rodrigáñez M. A., Díaz M., Tacon A. G. J. (2015). Application of In Vitro Digestibility Methods in Aquaculture: Constraints and Perspectives. *Reviews in Aquaculture*.

[31] Ezquerra J. M., García-Carreño F. L., Carrillo O. (1998). In Vitro Digestibility of Dietary Protein Sources for White Shrimp (Penaeus Vannamei). *Aquaculture*.

[32] Moyano F. J., Savoie L. (2001). Comparison of In Vitro Systems of Protein Digestion Using Either Mammal or Fish Proteolytic Enzymes. *Comparative Biochemistry and Physiology Part A: Molecular and Integrative Physiology*.

[33] Lemos D., Navarrete del Toro A., Córdova-Murueta J. H., Garcia-Carreño F. (2004). Testing Feeds and Feed Ingredients for Juvenile Pink Shrimp *Farfantepenaeus Paulensis*: In Vitro Determination of Protein Digestibility and Proteinase Inhibition. *Aquaculture*.

[34] Lemos D., Nunes A. J. P. (2008). Prediction of Culture Performance of Juvenile *Litopenaeus Vannamei* by In Vitro (pH-Stat) Degree of Feed Protein Hydrolysis With Species-Specific Enzymes. *Aquaculture Nutrition*.

[35] Tibbetts S. M., Yasumaru F., Lemos D. (2017). In Vitro Prediction of Digestible Protein Content of Marine Microalgae (Nannochloropsis Granulata) Meals for Pacific White Shrimp (*Litopenaeus Vannamei*) and Rainbow Trout (*Oncorhynchus Mykiss*). *Algal Research*.

[36] Lan C. C., Pan B. S. (1993). In Vitro Digestibility Simulating the Proteolysis of Feed Protein in the Midgut Gland of Grass Shrimp (*Penaeus Monodon*). *Aquaculture*.

[37] Buarque D. S., Castro P. F., Santos F. M. S., Lemos D., Júnior L. B. C., Bezerra R. S. (2009). Digestive Peptidases and Proteinases in the Midgut Gland of the Pink Shrimp *Farfantepenaeus Paulensis* (Crustacea, Decapoda, Penaeidae). *Aquaculture Research*.

[38] Kattakdad S., Jintasataporn O., Worawattanamateekul W., Chumkam S. (2018). pH Characterization of Digestive Enzyme and In Vitro Digestibility of Red Bee Shrimp Caridina Cantonensis (Decapoda: Atyidae). *Journal of Aquaculture Research and Development*.

[39] Divakaran S., Forster I. P., Velasco M. (2004). Limitations on the Use of Shrimp *Litopenaeus Vannamei* Midgut Gland Extract for the Measurement of In Vitro Protein Digestibility. *Aquaculture*.

[40] Bradford M. M. (1976). A Rapid and Sensitive Method for the Quantitation of Microgram Quantities of Protein Utilizing the Principle of Protein-Dye Binding. *Analytical Biochemistry*.

[41] Holm H., Hanssen L. E., Krogdahl Å., Florholmen J. (1988). High and Low Inhibitor Soybean Meals Affect Human Duodenal Proteinase Activity Differently: In Vivo Comparison With Bovine Serum Albumin. *The Journal of Nutrition*.

[42] Yasumaru F., Lemos D. (2014). Species Specific In Vitro Protein Digestion (pH-Stat) for Fish: Method Development and Application for Juvenile Rainbow Trout (*Oncorhynchus Mykiss*), Cobia (*Rachycentron Canadum*), and Nile Tilapia (*Oreochromis Niloticus*). *Aquaculture*.

[43] Rutherfurd S. M. (2010). Methodology for Determining Degree of Hydrolysis of Proteins in Hydrolysates: A Review. *Journal of AOAC International*.

[44] Adler-Nissen J. (1986). *Enzymatic Hydrolysis of Food Proteins*.

[45] De Muylder E., Lemos D., van der Velden G. (2008). Protein Hydrolysis of PAP Shows the Nutritive Value for Shrimp Feeds. *Aquaculture Asian Pacific*.

[46] Lemaire P., Bernard E., Martinez-Paz J. A., Chim L. (2002). Combined Effect of Temperature and Salinity on Osmoregulation of Juvenile and Subadult Penaeus Stylirostris. *Aquaculture*.

[47] Mariotti F., Tomé D., Mirand P. P. (2008). Converting Nitrogen Into Protein—Beyond 6.25 and Jones’ Factors. *Critical Reviews in Food Science and Nutrition*.

[48] Folch J., Lees M., Sloane Stanley G. H. (1957). A Simple Method for the Isolation and Purification of Total Lipides From Animal Tissues. *The Journal of Biological Chemistry*.

[49] Wilson R. P., Poe W. E. (1985). Effects of Feeding Soybean Meal With Varying Trypsin Inhibitor Activities on Growth of Fingerling Channel Catfish. *Aquaculture*.

[50] (1999). *Nutrition et Alimentation des Poissons et Crustacés.: Du Labo au Terrai*.

[51] Wickham H., Averick M., Bryan J. (2019). Welcome to the Tidyverse. *Journal of Open Source Software*.

[52] Kassambara A. (2016). CRAN: Contributed Packages.

[53] Kassambara A. (2019). CRAN: Contributed Packages.

[54] Komsta L. (2005). CRAN: Contributed Packages.

[55] Wood S. (2000). CRAN: Contributed Packages.

[56] Fox J., Weisberg S., Price B. (2001). CRAN: Contributed Packages.

[57] Müller K., Wickham H. (2016). CRAN: Contributed Packages.

[58] Wickham H., François R., Henry L., Müller K., Davis V. (2014). Dplyr: A Grammar of Data Manipulation.

[59] Cuzon G., Aquacop (2011). Nutritional Review of *Penaeus stylirostris*. *Reviews in Fisheries Science 6*.

[60] Wang S.-H., Chen J.-C. (2005). The Protective Effect of Chitin and Chitosan Against Vibrio Alginolyticus in White Shrimp *Litopenaeus Vannamei*. *Fish and Shellfish Immunology*.

[61] O’Brien J. J., Kumari S. S., Skinner D. M. (1991). Proteins of Crustacean Exoskeletons: I. Similarities and Differences Among Proteins of the Four Exoskeletal Layers of Four Brachyurans. *The Biological Bulletin*.

[62] Kuballa A., Abigail E. (2007). Novel Molecular Approach to Study Moulting in Crustaceans. *Bulletin of Fisheries Research Agency*.

[63] Rocha J., Garcia-Carreño F. L., Muhlia-Almazán A., Peregrino-Uriarte A. B., Yépiz-Plascencia G., Córdova-Murueta J. H. (2012). Cuticular Chitin Synthase and Chitinase mRNA of Whiteleg Shrimp *Litopenaeus Vannamei* During the Molting Cycle. *Aquaculture*.

[64] Richardson A., Dantas-Lima J., Lefranc M., Walraven M. (2021). Effect of a Black Soldier Fly Ingredient on the Growth Performance and Disease Resistance of Juvenile Pacific White Shrimp (*Litopenaeus Vannamei*). *Animals*.

[65] Zhang S.-P., Li J.-F., Wu X.-C. (2013). Effects of Different Dietary Lipid Level on the Growth, Survival and Immune-Relating Genes Expression in Pacific White Shrimp, *Litopenaeus Vannamei*. *Fish and Shellfish Immunology*.

[66] Ayisi C. L., Hua X., Apraku A., Afriyie G., Kyei B. A. (2017). Recent Studies Toward the Development of Practical Diets for Shrimp and Their Nutritional Requirements. *HAYATI Journal of Biosciences*.

[67] Li Y., Hu M., McClements D. J. (2011). Factors Affecting Lipase Digestibility of Emulsified Lipids Using an In Vitro Digestion Model: Proposal for a Standardised pH-Stat Method. *Food Chemistry*.

[68] Mat D. J. L., Souchon I., Michon C., Le Feunteun S. (2020). Gastro-Intestinal In Vitro Digestions of Protein Emulsions Monitored by pH-Stat: Influence of Structural Properties and Interplay Between Proteolysis and Lipolysis. *Food Chemistry*.

[69] Cousin M., Cuzon Gérard, Guillaume J., Aquacop (1996). Digestibility of Starch in Penaeus Vannamei: In Vivo and In Vitro Study on Eight Samples of Various Origin. *Aquaculture*.

[70] Silva B. C., Nolasco-Soria H., Magallón-Barajas F., Civera-Cerecedo R., Casillas-Hernández R., Seiffert W. (2016). Improved Digestion and Initial Performance of Whiteleg Shrimp Using Organic Salt Supplements. *Aquaculture Nutrition*.

[71] Lazo J. P., Romaire R. P., Reigh R. C. (1998). Evaluation of Three, In Vitro, Enzyme Assays for Estimating Protein Digestibility in the Pacific White Shrimp *Penaeus Vannamei*. *Journal of the World Aquaculture Society*.

[72] Satterlee L. D., Marshall H. F., Tennyson J. M. (1979). Measuring Protein Quality. *Journal of the American Oil Chemists’ Society*.

[73] Gauthier S. F., Vachon C., Jones J. D., Savoie L. (1982). Assessment of Protein Digestibility by In Vitro Enzymatic Hydrolysis With Simultaneous Dialysis. *The Journal of Nutrition*.

[74] Dimes L. E., Haard N. F. (1994). Estimation of Protein Digestibility—I. Development of an In Vitro Method for Estimating Protein Digestibility in Salmonids (Salmo gairdneri). *Comparative Biochemistry and Physiology Part A: Physiology*.

[75] Clark D. J., Lawrence A. L., Swakon D. H. D. (1993). Apparent Chitin Digestibility in Penaeid Shrimp. *Aquaculture*.

[76] Shiau S.-Y., Yu Y.-P. (1998). Chitin but Not Chitosan Supplementation Enhances Growth of Grass Shrimp, *Penaeus Monodon*. *The Journal of Nutrition*.

[77] Akiyama D. M., Coelho S. R., Lawrence A. L., Robinson E. H. (1989). Apparent Digestibility of Feedstuffs by the Marine Shrimp Penaeus Vannamei BOONE. *NIPPON SUISAN GAKKAISHI*.

[78] Ezquerra J. M., García-Carreño F. L., Civera R., Haard N. F. (1997). pH-Stat Method to Predict Protein Digestibility in White Shrimp (Penaeus vannamei). *Aquaculture*.

